# Must We Curtail Our Work?

**Published:** 1904-02

**Authors:** O. Egerton Schmidt

**Affiliations:** President


### CORRESPONDENCE.

### MUST WE CURTAIL OUR WORK?

The New York Orthopedic Dispensary and Hospital,
126 East 59th Street, New York City.

O. Egerton Schmidt, President.

January 19, 1904.

*Atlanta Journal-Record of Medicine, Atlanta, Ga.*

Gentlemen: During the last fiscal year the expenses of the New York Orthopedic Dispensary and Hospital have exceeded the receipts by $9,000. The total floating indebtedness of the Institution now amounts to $17,000. The Trustees of the Institution are not willing to continue the work of the Dispensary and Hospital upon the present basis without receiving a more hearty and general support from the public. I appeal to you, therefore, to come to our assistance. You may help the Institution in one of two ways:

I. An effort is being made to establish an Endowment Fund, the income from which will be used to make up the annual deficit, which usually amounts to from $5,000 to $10,000 in excess of such sums as may be received from portrait shows and other forms of entertainment. Subscriptions of $1,000 upward are receivable toward this fund. A good beginning has already been made, two subscriptions of $25,000 each having been received. Will you not help us in this matter?

II. If you do not see your way clear to subscribing to the Endowment Fund, will you not consent to become an annual subscriber? If the amount of your subscription is only $10, it will be thankfully received.

The Trustees of the Institution believe that they have a claim upon your attention and upon your assistance. During the fiscal year ending September 30, 1903, 3,879 patients were treated in our

Dispensary. Twenty-two thousand three hundred and eighty-five visits were made by patients to the Dispensary, an average of 80 a day. Five hundred and seven visits were made by surgeons to the homes of patients, and over 2,982 were made by nurses to patients at their homes, making a total of over 25,874 visits.

The expense of making instruments for the use of patients is a very serious item. The large cost of running the machine-shop where these instruments are made is only partially defrayed by the fees received from patients for the instruments. All are expected to pay for the instruments used, but payment is only exacted from such as are able to bear the expense. An average of $10 per year per patient would be a moderate allowance for the cost of apparatus, but for this amount we run our entire Hospital and Dispensary, including all the expenses for our surgeons, nurses, employees and machine-shop, and the expense account can not be further reduced.

A CRISIS.

Two courses are now open to the Board of Trustees as affecting the future policy of the Hospital. Either this splendid work which has been carried on by the Hospital for so long must be curtailed and the patients must be turned away to languish and die from the disabilities which can only be cured in their early youth, or else the number of subscribers to the work of the Hospital must be very largely increased.

Please give this matter your serious consideration. Do not throw this appeal to the waste-basket without giving the matter careful thought. The expenses of the Hospital have been reduced to the lowest possible point of economy, and no retrenchment can be made in this direction. Either the work must be curtailed or more annual subscribers must enroll their names on our list.

Yours faithfully,

O. EGERTON SCHMIDT,

President.

